# Combining creatinine and volume kinetics identifies missed cases of acute kidney injury following cardiac arrest

**DOI:** 10.1186/cc11931

**Published:** 2013-01-17

**Authors:** John W Pickering, Azrina Md Ralib, Zoltán H Endre

**Affiliations:** 1 Christchurch Kidney Research Group, Department of Medicine, University of Otago, P.O. Box 4345, Christchurch 8140, New Zealand; 2 Prince of Wales Hospital and Clinical School, University of New South Wales, High Street, Randwick, Sydney NSW 2031, Australia

## Abstract

**Introduction:**

Fluid resuscitation in the critically ill often results in a positive fluid balance, potentially diluting the serum creatinine concentration and delaying diagnosis of acute kidney injury (AKI).

**Methods:**

Dilution during AKI was quantified by combining creatinine and volume kinetics to account for fluid type, and rates of fluid infusion and urine output. The model was refined using simulated patients receiving crystalloids or colloids under four glomerular filtration rate (GFR) change scenarios and then applied to a cohort of critically ill patients following cardiac arrest.

**Results:**

The creatinine concentration decreased during six hours of fluid infusion at 1 litre-per-hour in simulated patients, irrespective of fluid type or extent of change in GFR (from 0% to 67% reduction). This delayed diagnosis of AKI by 2 to 9 hours. Crystalloids reduced creatinine concentration by 11 to 19% whereas colloids reduced concentration by 36 to 43%. The greatest reduction was at the end of the infusion period. Fluid dilution alone could not explain the rapid reduction of plasma creatinine concentration observed in 39 of 49 patients after cardiac arrest. Additional loss of creatinine production could account for those changes. AKI was suggested in six patients demonstrating little change in creatinine, since a 52 ± 13% reduction in GFR was required after accounting for fluid dilution and reduced creatinine production. Increased injury biomarkers within a few hours of cardiac arrest, including urinary cystatin C and plasma and urinary Neutrophil-Gelatinase-Associated-Lipocalin (biomarker-positive, creatinine-negative patients) also indicated AKI in these patients.

**Conclusions:**

Creatinine and volume kinetics combined to quantify GFR loss, even in the absence of an increase in creatinine. The model improved disease severity estimation, and demonstrated that diagnostic delays due to dilution are minimally affected by fluid type. Creatinine sampling should be delayed at least one hour following a large fluid bolus to avoid dilution. Unchanged plasma creatinine post cardiac arrest signifies renal injury and loss of function.

**Trial registration:**

Australian and New Zealand Clinical Trials Registry ACTRN12610001012066.

## Introduction

Resuscitation of intravascular volume of critically ill patients is essential for hemodynamic support [1-3]. Accurate estimates of kidney function remain elusive in the absence of real-time measurement of the glomerular filtration rate (GFR). Plasma creatinine remains the default surrogate biomarker of GFR [4-6]. Dilution of plasma creatinine by fluid loading [7] is usually not considered. However, if fluid resuscitation dilutes plasma creatinine, the incidence of acute kidney injury (AKI) may be under-reported, diagnosis and intervention delayed, and GFR overestimated.

Creatinine kinetic modeling and back-calculating baseline creatinine from presumed GFR have evaluated AKI definitions [5,8,9], identified creatinine-based outcome metrics for clinical trials [10], quantified the effect of calculated baseline creatinine on AKI epidemiology [11-13], and compared methods of normalizing urinary injury biomarkers [14,15]. None of these models incorporates the dilution effect of fluid loading on plasma creatinine concentration, nor do they allow for dynamic changes in creatinine generation.

Volume kinetic modeling is used to simulate the distribution and clearance of infused fluids [16]. It has been used to investigate the differences in distribution of colloids and crystalloids in volunteers, the influence of anesthesia and surgery on clearance [17,18], and patient response to crystalloid infusion (for example, in women with pre-eclampsia) [19].

We developed a model that combined volume and creatinine kinetics. The model was first applied to simulated patients to assess the extent of plasma creatinine concentration change due to fluid loading and its effect on AKI diagnosis. We then used the model to assess the changes in renal function and creatinine generation in a cohort of 49 cardiac arrest patients. The limitations of the model were further assessed by a detailed examination in three patients.

## Materials and methods

A two-compartment creatinine kinetic model and a two-compartment volume kinetic model were combined into a volume-creatinine kinetic model. The initial conditions of the model were assessed in a simulated patient. The model was then tested in a cohort of patients resuscitated after cardiac surgery. Three patients were presented as case studies. Data collection was approved by the Upper South A Regional ethics committee of New Zealand (URA/09/09/062). Screening on entry to the emergency department (ED) was by presumptive consent, followed by written consent from the patient or family.

### Creatinine kinetics

**Figure 1:**
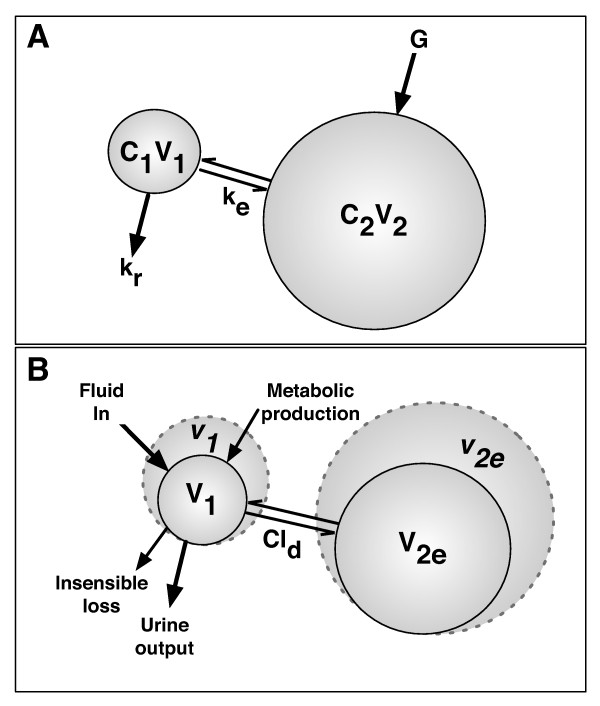
**Creatinine kinetic model (a) and volume kinetic model (b)**. C_1 _and C_2_, creatinine concentrations of the plasma (1) and extracellular (2) compartments, respectively; Cl_d_, distribution clearance; G, creatinine generation rate; k_e_, rate constant for creatinine diffusion between compartments; k_r_, rate constant for creatinine excretion; *v*, expanded volume; V_1 _and V_2_, creatinine volumes of the plasma (1) and extracellular (2) compartments, respectively; V_2e_, volume of the expandable compartment.

The creatinine kinetic model is shown in Figure 1a. Creatinine generated in muscle cells enters the extravascular compartment (*V*_2_) at a rate *Ġ*, is freely exchanged with the plasma compartment (*V*_1_), and is eliminated by the kidney at a rate determined by the rate constant, *k_r _*(note: *GFR *= *k_r_V*_10_). We used dot notation for differentiation (for example, $Ġ=\frac{dG}{dt}$) and a subscript '0' to represent the value of the variable at time zero.

Given that the total volume of distribution of creatinine (*V*_1 _+ *V*_2_) is equal to the total body water, the extravascular volume is equal to the total body water minus the plasma volume; it is assumed that creatinine is not bound and flows freely from one compartment with the same permeability as water. The rate of change of mass in each compartment was determined by mass balance (Table 1). Because creatinine was not bound, the rate of exchange between compartments (*k_e_*) was the same in both directions. Conditions at equilibrium, where there is zero rate of change of creatinine, creatinine production, and compartment volumes, were used to ascertain initial conditions for *k_r _*and *k_d _*(see Additional file 1 containing extended methods).

**Table 1:** Creatinine and volume kinetic components

Compartment	Rate of change of mass/volume	Gain	Losses
Creatinine 1	${Ċ}_{1}V_{1}+C_{1}{\dot{V}}_{1}$	*k_e_C*_2_*V*_2_	*k_e_C*_1_*V*_1 _+ *k_r_C*_1_*V*_1_

Creatinine 2	$C_{2}{\dot{V}}_{2}+{Ċ}_{2}V_{2}$	*Ġ *+ *k_e_C*_1_*V*_1_	*k_e_C*_2_*V*_2_

Plasma, 1	${\dot{V}}_{1}$	*Ḃ *+ *Ṁ*	$İ+\dot{U}+{\dot{V}}_{2e}$

Expandable, 2e	${\dot{V}}_{2e}$	$Cl_{d}\frac{\left(v_{1}-v_{10}\right)}{v_{10}}$	$Cl_{d}\frac{\left(v_{2e}-v_{2e0}\right)}{v_{2e0}}$

Note: dot notation was used for differentiation (for example, $Ċ=\frac{dC}{dt}$). *C*_1 _and *C*_2_, creatinine concentrations for compartments 1 and 2, respectively. *v_c _*and *v_t_*, time-varying volumes; *V*_1 _and *V*_2_, creatinine volumes for compartments 1 and 2, respectively. *V*_10 _and *V*_2*e*0_, time zero compartment volumes.

### Volume kinetics

Volume kinetics simulates the distribution and elimination of infused fluids and usually involves two compartments: one approximates the plasma volume (*V*_10 _prior to expansion) and the other approximates the expandable component of the interstitial fluid space (*V_t_*0 prior to expansion) [16] (Figure 1b). *V*_2*e*0 _is smaller than the interstitial space because expansion of body fluids is not possible in all regions (for example, in the skeleton) [16]. As fluid is infused into the vasculature, the plasma compartment volume will expand at a rate dependent on the rate of infusion (*Ḃ*), metabolic production (*Ṁ*), insensible loss (*İ*), urine output $\left(\dot{U}\right),$ and exchange between compartments. The rate of exchange between compartments depends on the distribution clearance (*Cl_d_*) and the relative differences in the expansion of the two volumes (Table 1). We assume that water can flow freely in both directions between compartments [16] and that there is no significant loss to a third space.

### Combined creatinine kinetic and volume kinetic model

The creatinine and volume kinetic models were combined assuming that creatinine kinetic plasma compartment and volume kinetic plasma volume were the same [16]. The extravascular creatinine compartment volume (*V*_2_) is equal to the sum of the expandable (*v*_2*e*_) and non-expandable (*V*_2*n*0_) fluid volumes: *V*_2 _= *v*_2*e *_+ *V*_2*n*0_; therefore, ${\dot{V}}_{2}={\dot{V}}_{2e}.V_{2n0}$ equals the difference between the initial extravascular and expandable interstitial space volumes (*V*_2*n*0 _= *V*_20 _- *V*_2*e*0_). The model was programmed in Matlab (Matlab 2011b; MathWorks Inc., Natick, MA, USA) and solved numerically with the ordinary differential equation solver *ode45*. Extended methods are presented in Additional file 1.

### The simulated patient

#### Effect of fluid loading and differential rates of urine output

The change in creatinine concentration and fluid balance as a function of time was calculated for a 70-kg male with total body water of 42 L, plasma volume of 2.8 L, expandable interstitial volume of 8.4 L [16], baseline GFR of 100 mL/minute, baseline creatinine concentration of 1 mg/dL, and initial creatinine generation rate of 1 mg/minute, which decreased by 1.5% per day [20,21]. Simulated patient demographics are presented in Table 2.

**Table 2:** Simulated virtual-in-patient demographics

Ideal total body water (ITBW), mL	42,000
Initial plasma volume (*V*_10_), mL	2,800 (ITBW/15)

Initial expandable space volume (*V*_2*e*0_), mL	8,400 (3 × ITBW/15)

Initial creatinine generation rate (*Ġ*_0_), mg/minute	1

Glomerular filtration rates	100, 66.7, 50, 33.3

Insensible water loss rate (*İ*), mL/day	800

Metabolic water generation rate (*Ṁ*), mL/day	400

Distribution clearance rate (*Cl_d_*), mL/minute	200, 10

Initial plasma creatinine concentrations (*C*_10 _≡ *C*_20_), mg/dL	1

#### Urine output

The rate of urine output depends on hydration status. In someone with normal kidney function, extreme hypertonic dehydration (loss of 10% of total body water) results in approximately a 70% reduction in urine output [22,23], whereas overhydration will produce a rapid increase in urine output, peaking at approximately 900 mL/hour. These initial conditions were used to develop a formula to describe change in urine output (Additional file 1). Failure of reabsorption results in polyuric AKI, which was not modeled.

#### Fluid input

Two fluid input scenarios were modeled: (a) maintenance fluid alone and (b) maintenance + boluses of either crystalloids or colloids of 1 L per hour for 6 hours beginning at the end of hour one. This represents a high fluid input and corresponded to the 80th percentile of the fluid input over the course of the first 6 hours of the patients in the cardiac arrest cohort. For each fluid input scenario, there were four GFR scenarios: no change in GFR and a decrease by one third, one half, or two thirds at the time of insult (t = 1 hour). These correspond to GFR criteria for RIFLE AKI severity stages R (Risk), I (Injury), and F (Failure). On the basis of creatinine kinetic modeling alone, these produce creatinine increases of 50%, 100%, and 200%, respectively [9].

The crystalloid distribution exchange rate (*Cl_d_*) was set at 200 mL/minute. This was based on multiple studies with Ringer's lactate (Table S3 in Additional file 1). Colloids have much longer half-lives in the vasculature [1,24,25] and correspondingly smaller distribution exchange rates. *Cl_d _*for colloids was set at 10 mL/minute.

Metabolic water production was assumed to be 400 mL/day (16.7 mL/hour), and insensible losses 800 mL/day (33.4 mL/hour). At a GFR of 100 mL/minute followed by 99% reabsorption, urine output was 1 mL/minute (60 mL/hour). Therefore, maintenance fluid was set at 76.7 mL/hour $\left(\text{maintenance}=\dot{U}+\left(İ-Ṁ\right)\right)$.

#### Sensitivity analysis

The sensitivity of the model was assessed for differences in *Cl_d _*(50, 100, 300, and 400 mL/minute compared with 200 mL control), the difference between insensible loss and metabolic water production (0 and 800 mL compared with 400 mL control), the ratio of the plasma volume to volume of expandable interstitial space (1:2 and 1:4 ratios compared with 1:3 control), and a loss or gain of creatinine production for the duration of the illness (30%).

### Patient cohort

Patients admitted following cardiac arrest were a subcohort from the Early Detection of Acute Kidney Injury (EDAKI) study conducted in the ED and intensive care unit of Christchurch Hospital (Christchurch, New Zealand). Ethics approval was obtained from the Upper South A Regional Ethics Committee (URA/09/09/062), and informed consent was obtained from patients or relatives.

After ICU admission, each patient immediately underwent therapeutic hypothermia for 24 hours (core temperature reduced to 33°C). The resuscitation fluid was 0.9% saline in each case prior to arrival at the ED. Hourly urine output and fluid input were recorded for each patient. Plasma samples were collected on admission to the ED, on admission to the ICU, and at least daily thereafter. Urine samples for measurement of neutrophil gelatinase-associated lipocalin (NGAL), cystatin C, alkaline phosphatase (ALP), gamma-glutamyltranspeptidase (GGT), and α- and π-glutahione-S-transferase (α- and π-GST) were taken on catheterization in the ED, on admission to the ICU, and 4, 8, and 16 hours later and daily.

To assist assessment, patients were divided into three groups: Cr_increase _(a plasma creatinine increase of more than 20% at 24 hours after cardiac arrest), Cr_decrease _(a plasma creatinine decrease of more than 10% at 24 hours), and Cr_unchanged _(neither Cr_increase _nor Cr_decrease_). The first male enrolled in each group was selected as a case study:

Patient A was a 90-kg male with a history of hypertrophic obstructive cardiomyopathy and with severely impaired left ventricular function (ejection fraction of 25%). He had presented to the ED, complaining of abdominal pain, nausea, and vomiting. His condition deteriorated, requiring intubation, during which time he suffered a cardiac arrest followed by cardiopulmonary resuscitation (CPR). He was defibrillated twice (at 360 J), and adrenaline was given. After 50 minutes, he regained cardiac output and was transferred to the ICU. Over the next 4 days, his cardiopulmonary status improved steadily; however, presumably owing to hypoxic brain injury, there was no neurological recovery. Therapy was withdrawn and he died.

Patient B was an 87-kg male with a history of alcoholism. He collapsed during a shower. Resuscitation time was 20 minutes with marginal CPR. He received 1.5 L of normal saline in the ED and 1 L in the first two hours in the ICU. Prognosis was assessed as poor after rewarming. Multiple fluid boluses were given, but the patient became oliguric and died 53 hours after arrest.

Patient C was an 80-kg male with no medical history. He suffered a cardiac arrest during a motor vehicle inspection. Immediate CPR was performed by a nurse bystander before the arrival of paramedics. Resuscitation lasted 27 minutes with defibrillation (once) and two adrenaline boluses. An electrocardiograph showed an inferolateral myocardial infarct, and the patient underwent emergency percutaneous coronary intervention with a stent inserted to repair the left circumflex artery, which was completely occluded. He was transferred to the ICU for cooling. There were several occasions of bradycardia needing boluses of atropine and later adrenaline infusion. After rewarming, his condition improved and he was extubated. He was discharged to a coronary care unit on day 3. Model application to the cardiac arrest patients used the measured urine output from each patient rather than the simulated urine output of the simulated patient.

## Results

### Model application in simulated patients

#### Glomerular filtration rate maintained

**Figure 2:**
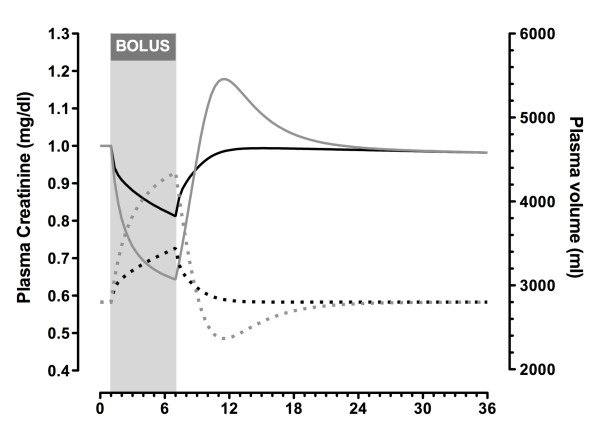
**Simulated changes in plasma creatinine and plasma volume while glomerular filtration rate was maintained**. Plasma volume (dotted lines) and plasma creatinine (solid lines) in a 70-kg male (total body water of 42 L) according to a plasma creatinine generation rate of 1 mg/minute and a baseline plasma creatinine of 1 mg/dL (equivalent to a creatinine clearance of 100 mL/minute) after an infusion of crystalloid (black lines) or colloid (grey lines) at 1 L/hour for 6 hours.

Creatinine concentration decreased as fluid was infused, and plasma volume increased while GFR was held constant (Figure 2). The minimum concentration at the end of the infusion was 19% below the starting concentration for the crystalloid infusion and 36% below for the colloid infusion. The concentration returned to normal within about 6 hours after crystalloid infusion but took an additional approximately 10 hours after colloid infusion. Colloid infusion produced an increase in creatinine concentration above baseline from 2 to approximately 16 hours following infusion. During this period, renal elimination of excess body water occurred at a rate greater than the flow of water from the extravascular space into the plasma, resulting in a brief decrease in plasma volume. Urine output peaked at 825 mL/hour at the end of the fluid bolus, at which time the simulated patient was 5.7% overhydrated. It required a further 6 hours before urine output decreased below 100 mL/hour.

#### Glomerular filtration rate decreased

**Figure 3:**
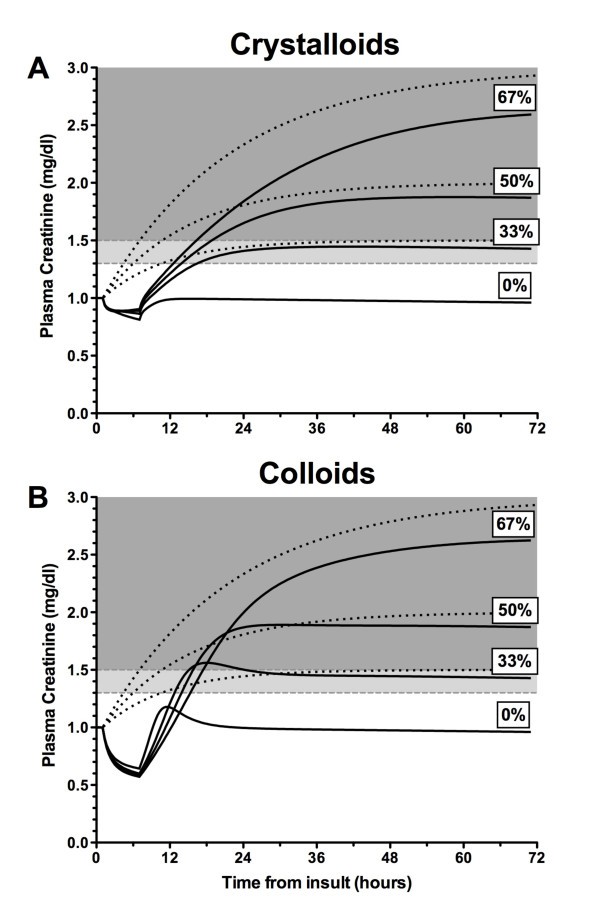
**Simulated changes in plasma creatinine in the presence of reduced glomerular filtration rate (GFR)**. Plasma creatinine in a 70-kg male according to a plasma creatinine generation rate of 1 mg/minute and a baseline plasma creatinine of 1 mg/dL. Values are shown following an infusion of crystalloid **(a) **or colloid **(b) **at 1 L/hour for 6 hours. In each scenario, the creatinine curves (dark lines) are plotted for no loss (0%) and 33.3%, 50%, and 66.7% loss of GFR (step change) at t = 1 hour. Dotted lines represent the corresponding theoretical change in creatinine if there was no accounting for fluid balance or the 1.5%/day creatinine production reduction due to muscle wasting. Dark shading represents acute kidney injury (AKI) by the RIFLE (Risk, Injury, Failure, Loss, and End-stage kidney disease) definition (> 50% increase in plasma creatinine above baseline), and light shading represents AKI by the AKIN (Acute Kidney Injury Network) definition alone (> 0.3 mg/dL increase in plasma creatinine above baseline).

Even with concomitant loss of GFR, there was a reduction in plasma creatinine during fluid infusion. This reduction was similar irrespective of the degree of GFR decrease. After fluid administration, the increase in creatinine was smaller than expected in AKI based on creatinine kinetic modeling alone (Figure 3), except for a brief period following colloid infusion when there was a transient increase in creatinine above that predicted by creatinine kinetics alone. Diagnosis of AKI was delayed by 2 to 9 hours by administration of fluids, depending on the extent of loss of GFR and type of fluid administered.

#### Sensitivity analysis

A sensitivity analysis of input variables was performed for patients receiving 6 L of crystalloid the course of 6 hours with no change in GFR. The model was insensitive to differences in the rate of water distribution, ranging from 50 to 400 mL/minute, with a maximum difference in plasma creatinine concentration of 8.9%. The model was even less sensitive to a ± 400 mL/day difference between insensible loss and metabolic water production, with a maximum difference of 4.7%. Similarly, the model was insensitive to variations in the ratio of the plasma to expandable space (*V*_10 _to *V*_*e*0_), with maximum differences in creatinine concentration from the 1:3 *V*_10 _to *V*_*e*0_ ratio of -5.5% (1:2 ratio) and 3.6% (1:4). A decrease of 30% in creatinine generation maintained for 72 hours led to a 30% decrease in plasma concentration (Additional file 1).

### Model application to patients after cardiac arrest

**Figure 4:**
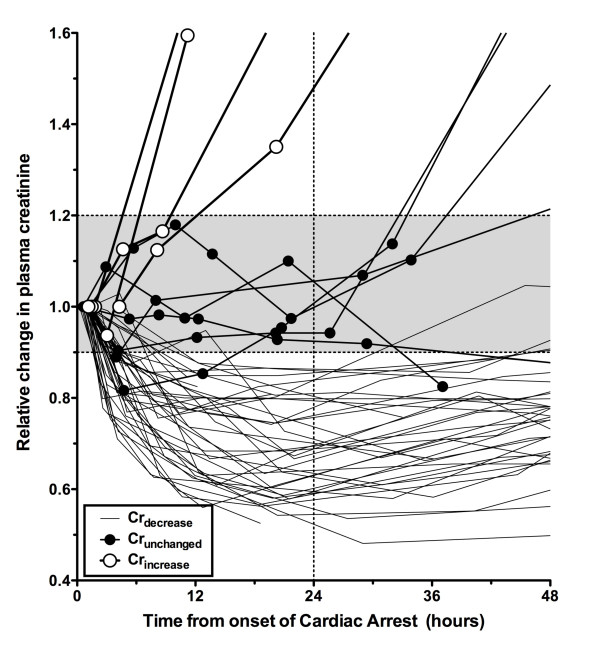
**Changes in plasma creatinine concentration relative to the first creatinine measured in the emergency department followed one of three patterns**. These patterns are Cr_increase _(open white circles), in which the increase in plasma creatinine was greater than 20% at 24 hours after cardiac arrest; Cr_unchanged _(closed solid circles), in which plasma creatinine increased no more than 20% or decreased no more than 10% at 24 hours after cardiac arrest, and Cr_decrease_, in which plasma creatinine decreased exponentially by more than 10% by 24 hours after cardiac arrest.

Forty-nine patients were enrolled on entry to the ED. Patient demographics and initial parameters are shown in Table 3 for all 49 patients and in Table 4 with additional model parameters for the three case studies. Three main patterns of change in creatinine concentration were observed during the first 24 hours: a rapid increase (Cr_increase_: *n *= 4), little change (Cr_unchanged_: *n *= 6), or a rapid decrease (Cr_decrease_: *n *= 39) (Figure 4). Cr_increase _patients were all designated as clinical AKI by the Acute Kidney Injury Network (AKIN) definition (> 0.3 mg/dL increase in plasma creatinine within 48 hours). The Cr_unchanged _group showed fluctuations between a 20% increase and a 10% decrease in creatinine relative to the initial ED concentration. Patients had a relative reduction of 14% to 47% (median 32%, IQR 21% to 39%) within 24 hours. In the Cr_decrease _group, the plasma cystatin C concentrations also had a relative reduction of 6% to 41% (median 37%, IQR 28% to 43%), and urinary creatinine concentrations were low or rapidly decreased. All in the Cr_increase_, 5 of 6 Cr_unchanged_, and 36 of 39 Cr_decrease _groups had urine outputs of less than 0.5 mL/hour per kg for at least 6 hours for any 6-hour period during the first 48 hours (AKIN urine output AKI criterion).

**Table 3:** Cardiac arrest patient demographics (*n *= 49)

Demographic	Value
Male	38 (78)

Age, years	61 (16)

Weight, kg	80 (13)

APACHE II score	20 (4.4)

SOFA score	8.1 (2.3)

Sepsis	4 (8)

Mechanical ventilation	49 (100)

Cooling	46 (94)

Emergency PCI	15 (31)

ROSC duration, minutes	20 (12.5-30)

DC shock, number	3 (1-5)

Adrenaline, mg	1 (0-3)

First plasma sample	

B-type natriutetic peptide, pg/mL	64 (19-194)

Creatinine kinase, U/L	365 (185-920)

Troponin, μg/L	0.05 (0.03-0.175)

Creatinine, mg/dL	1.21 (1.11-1.37)

Cystatin C, mg/L	0.93 (0.77-1.11)

Urea, mmol/L	6.6 (5.4-7.8)

Outcomes	

Renal replacement therapy	3 (6)

Death	23 (47%)

ICU LOS, hours	66 (48-103)

Hospital LOS, days	8.7 (3.9-14)

Data are presented as number (percentage), mean (standard deviation), or median (lower quartile to upper quartile). APACHE II, Acute Physiology and Chronic Health Evaluation II; DC, Direct Current; ICU, intensive care unit; LOS, length of stay; PCI, percutaneous coronary intervention; ROSC, return of spontaneous circulation; SOFA, Sequential Organ Failure Assessment.

**Table 4:** Demographic and model parameters applied to case studies

Parameter	Equations	Case A	Case B	Case C
Sex		Male	Male	Male

Weight, kg		90	87	80

APACHE II score		26	21	19

SOFA score		12	8	4

ROSC duration, minutes		50	20	27

Time from arrest to first in-hospital plasma creatinine, minutes		-91^a^	84	26

Total body water (TBW), mL	60 × weight in kg	54,000	52,200	48,000

Plasma volume (*V*_10_), mL	TBW/15	3,600	3,480	3,200

Creatinine compartment outside of plasma (*V*_20_)	TBW - plasma volume	50,400	48,720	44,800

Expandable space [16] (*V*_2*e*0_), mL	Plasma volume × 3	10,800	10,740	9,600

Insensible loss rate (*İ*), mL/minute	(800 mL/day)	0.56		0.56

Metabolic production rate (*Ṁ*), mL/minute	Insensible loss rate/2	0.28		0.28

Baseline creatinine production rate [38] (*Ġ*), mg/minute	(27 - 0.173 × age in years) × weight in kg/1,440	1.43		0.885

Distribution clearance (*Cl_d_*), mL/minute		200	200	200

^a^As this patient arrested in the emergency department (ED), a baseline creatinine was available on entry to the ED, 91 minutes prior to cardiac arrest. APACHE II, Acute Physiology and Chronic Health Evaluation II; ROSC, return of spontaneous circulation; SOFA, Sequential Organ Failure Assessment.

### Creatinine decrease (Cr_decrease_)

**Figure 5:**
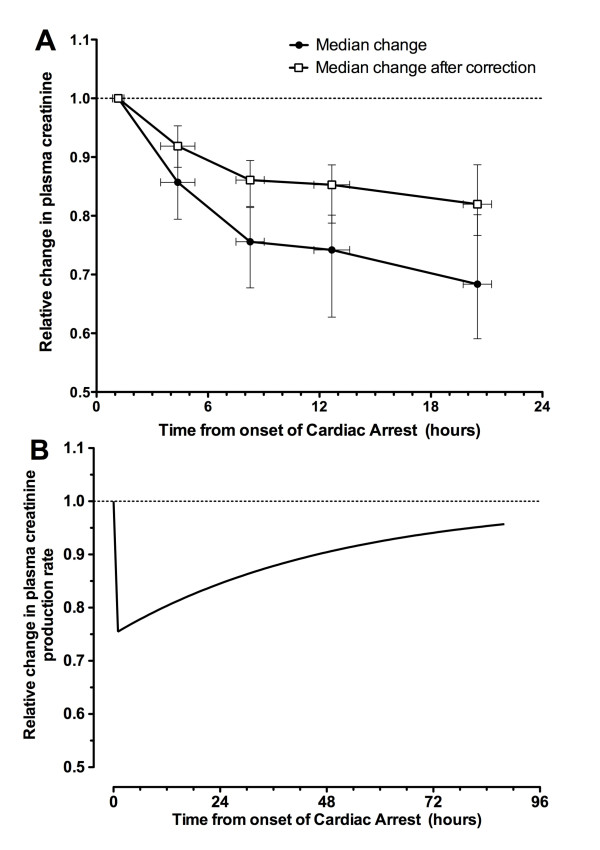
**Changes in plasma creatinine concentration in the Cr_decrease _patients**. **(a) **Median change in plasma creatinine concentration relative to the first creatinine measured in the emergency department for Cr_decrease _patients. Solid circles represent raw changes. Open white squares show changes after correction for fluid input and urine output by using the volume-creatinine kinetic model and assuming maintenance of glomerular filtration rate. Whiskers show interquartile range. **(b) **The median changes in creatinine production necessary to account for the observed reduction in plasma creatinine shown in panel (a). Cr_decrease_, group of patients with a plasma creatinine decrease of more than 10% at 24 hours after cardiac arrest.

Applying the volume-creatinine kinetic model while assuming no loss of GFR showed that dilution could explain 43% (IQR 26% to 67%) of the maximum reduction in creatinine concentration in the Cr_decrease _patients within the first 24 hours. The remaining reduction in creatinine concentration may be explained and modeled as a loss of creatinine production (Figure 5) or an increase in GFR assuming patients had AKI on admission.

#### Creatinine unchanged (Cr_unchanged_)

**Figure 6:**
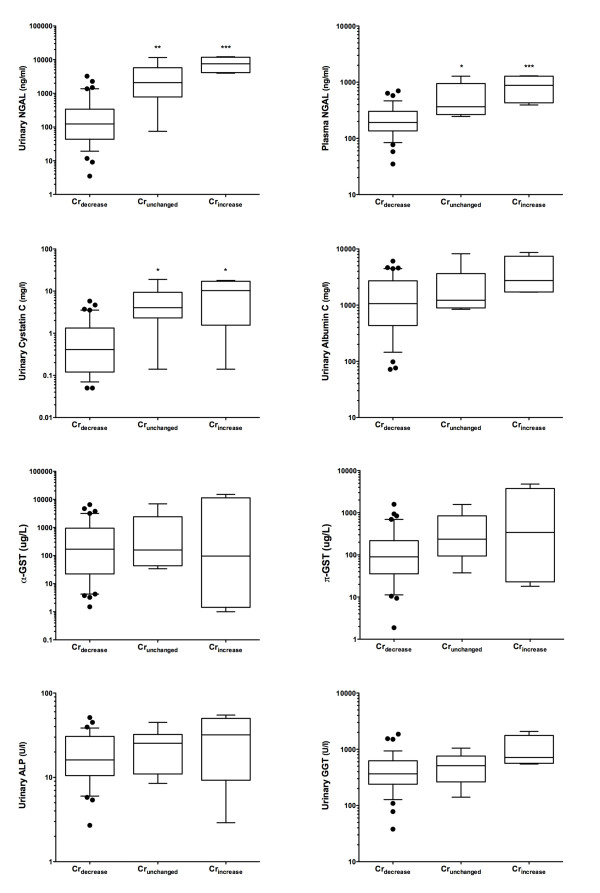
**Kidney injury biomarkers associated with Cr_increase _and Cr_unchanged _groups**. Urinary and plasma neutrophil gelatinase-associated lipocalin (NGAL) (*P *< 0.0001) and urinary cystatin C (*P *= 0.0017) showed an increasing trend, and both Cr_unchanged _and Cr_increase _were significantly different from Cr_decrease _(*P < 0.05, **P < 0.01, ***P < 0.001, Tukey's multiple comparison test). Apparent trends in urinary albumin, gamma-glutamyltranspeptidase (GGT), and π-glutahione-S-transferase (π-GST), the other biomarkers, were at a *P *value of greater than 0.05. Whiskers are at 10th and 90th percentiles. Cr_increase_, group of patients with a plasma creatinine increase of more than 20% at 24 hours after cardiac arrest; Cr_unchanged_, group of patients in whom plasma creatinine increased no more than 20% or decreased no more than 10% at 24 hours after cardiac arrest.

Over the course of the first 24 hours, a mean 52% (standard deviation 13%) decrease in GFR was required to account for the lack of reduction in plasma creatinine taking into account fluid input and urine output and assuming a loss of creatinine production the same as the median loss for the Cr_decrease _cohort (Figure 5b). Multiple injury biomarkers were increased in the Cr_unchanged _patients: plasma NGAL (*P *< 0.01), urinary NGAL (*P *< 0.01), and urinary cystatin C (*P *< 0.01) compared with the Cr_decrease _cohort (Figure 6). All 10 of the Cr_increase _or Cr_unchanged _patients died within 30 days, whereas 13 of the 39 Cr_decrease _patients died (*P *< 0.0001).

### Case studies

#### Case A: Cr_increase_

AKI was detected 11 hours 46 minutes after cardiac arrest (plasma creatinine of 1.36 mg/dL compared with 0.67 mg/dL on admission). Plasma NGAL was elevated in the first sample 1:49 hours after arrest (286 ng/mL). Urinary NGAL (2,990 ng/mL), GGT (613 U/L), π-GST (38 μg/L), and albumin (2,900 mg/L) were elevated in the first urine sample (6:59 hours after arrest). The patient was oliguric (urine output of less than 0.5 mL/kg per hour) for 19 hours from 3 hours after arrest (AKIN stage 2) and had a 3-hour period of anuria from 29 hours.

**Figure 7:**
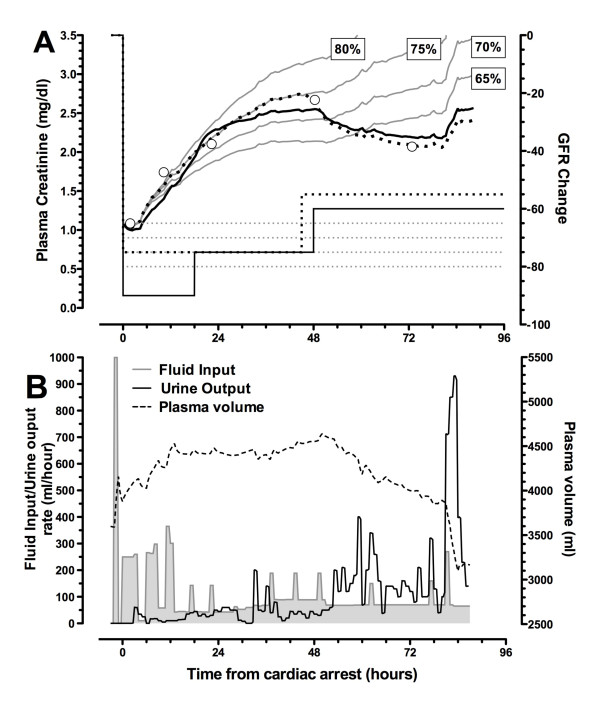
**Case A: Cr_increase_**. Effect of changing glomerular filtration rate (GFR) on calculated plasma creatinine is shown. **(a) **Plasma creatinine. Open circles represent measured plasma creatinine concentrations, and solid grey lines represent modeled plasma creatinine concentrations according to respective GFR decreases of 65%, 70%, 75%, and 85%. The dotted black line represents the best-fit modeled plasma creatinine that results from step changes in GFR shown at the bottom of panel (a) (right axis). The solid black line represents the best fit taking into account a loss of creatinine production, assumed to be the same as the median loss modeled for the Cr_decrease _group (Figure 4b), in addition to changes in GFR. This suggests that the measured creatinine values can be explained by an immediate 90% decrease in GFR followed by two step increases of approximately 15% in GFR with an initial decrease in creatinine production of 25%. **(b) **Measured fluid input (shaded area), measured urine output (solid line), and calculated plasma volume (dashed line). The rate of fluid input initially exceeded urine output, resulting in increases in total body water and in plasma volume. Plasma volume returned to normal toward the end of this period. Cr_increase_, group of patients with a plasma creatinine increase of more than 20% at 24 hours after cardiac arrest.

Creatinine change taking into account fluids given, urine output, and change in GFR was modeled (Figure 7a). A GFR decline of 75% improving to 55% after 48 hours best represented the observed creatinine concentrations with the minimum number of GFR changes (two). Including the median loss of creatinine determined from the Cr_decrease _patients (Figure 5b), the best-fit GFR showed an initial greater loss of GFR (90%). A short rapid rise in creatinine at the end of the observation period was due to a brief period of polyuria shortly prior to death (Figure 7b).

#### Case B: Cr_unchanged_

Plasma creatinine remained static for the first 24 hours before doubling prior to death. Urinary biomarkers were all elevated in the ED sample and increased, reaching a maximum 5 hours after arrest: NGAL 1,016 ng/mL, cystatin C 3.0 mg/L, GGT 1,050 U/L, α-GST 34 μg/L, and π-GST 143 μg/L. Plasma NGAL was also elevated in the first sample: 302 ng/mL. Urine output varied between 20 and 60 mL/hour during the first 18 hours. The patient then showed periods of oliguria during the ensuing 35 hours prior to death, despite large fluid boluses.

**Figure 8:**
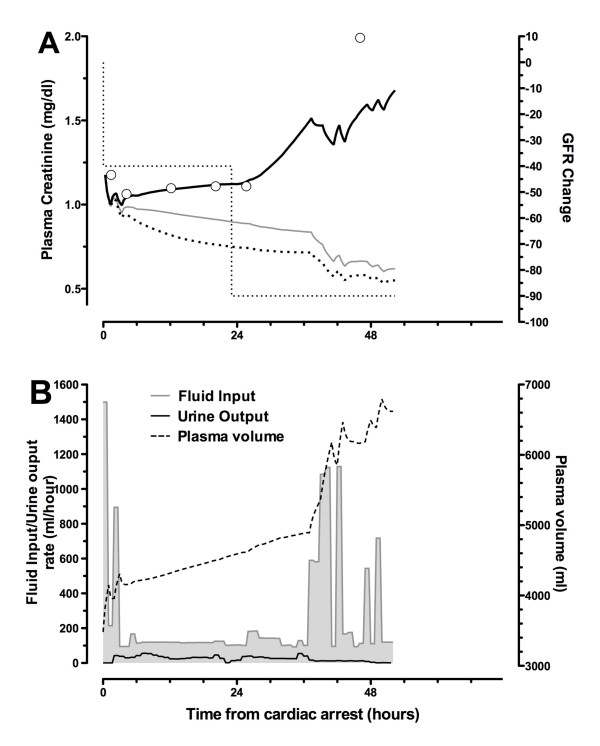
**Case B: Cr_unchanged_**. Plasma creatinine changes **(a) **and fluid input/urine output and plasma volume **(b) **are shown. Fluids alone (no change in glomerular filtration rate (GFR) and no reduction in creatinine production) suggest that a decrease in plasma creatinine should have been observed (grey line). If there had been a reduction in creatinine production (Figure 4), the decrease would have been greater (dotted line). A model including a reduction in creatinine, fluids in and out, and a reduction of 40% in GFR increasing to 90% at about 24 hours best fits the measured creatinine. The patient became anuric despite multiple boluses and died at 53 hours. Cr_unchanged_, group of patients in whom plasma creatinine increased no more than 20% or decreased no more than 10% at 24 hours after cardiac arrest.

During the first 24 hours, the retained fluid should have resulted in a decrease in creatinine concentration (grey line, Figure 8a). If creatinine production had also decreased, this would have increased the expected reduction in creatinine concentration (dotted line, Figure 8a). Failure to observe this reduction could be accounted for by a GFR decrease of 40% during this period. A further reduction after 24 hours accounted for the rapid increase in plasma creatinine.

#### Case C: Cr_decrease_

**Figure 9:**
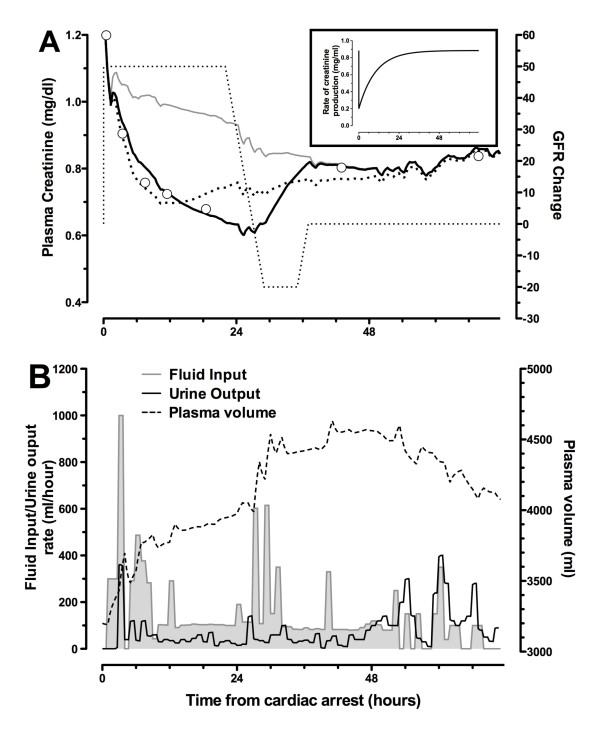
**Case C: Cr_decrease_**. Plasma creatinine changes **(a) **and fluid input/urine output and plasma volume **(b) **are shown. Fluids alone cannot account for the reduction in plasma creatinine (grey line). Either glomerular filtration rate (GFR) must be assumed to increase dramatically followed by a decrease (dotted or lines) or there is a rapid reduction in plasma creatinine production (black lines) (see 'Case C: Cr_decrease_' subheading). Cr_decrease_, group of patients with a plasma creatinine decrease of more than 10% at 24 hours after cardiac arrest.

The patient received three boluses of normal saline in the ED (totaling 1,600 mL) and four small boluses (250 to 450 mL) each hour for the first 4 hours in the ICU. Plasma creatinine decreased more rapidly than can be explained by dilution (Figure 9a). Urinary creatinine decreased from 15.2 mmol/L 50 minutes after arrest to 3.1 mmol/L 3:40 hours later. It did not recover to the earlier level until 42 hours after arrest. Injury biomarkers appeared briefly elevated in the first ED sample (NGAL 347 ng/mL, cystatin C 0.54 mg/L, GGT 900 U/L, α-GST 3,800 μg/L, and π-GST 56 μg/L) but rapidly returned to normal ranges by the first ICU sample 95 minutes later.

## Discussion

The combined volume and creatinine kinetic model clearly demonstrates that fluid resuscitation leads to an underestimation of plasma creatinine concentration and AKI severity or alternatively to diagnostic delay or failure to detect AKI. This strongly supports the use of an adjusted creatinine on the basis of fluid balance, as suggested by Macedo and colleagues [26]. The model shows that the extent to which plasma creatinine was underestimated depended on the rate of fluid infusion, the type of fluid (crystalloid or colloid), the timing of the plasma creatinine sample in relation to the timing of fluid infusion, and the rate at which excess fluid was excreted by the urine. The type of fluid minimally affected diagnosis delay in the scenario of 6 L of fluid given over the course of 6 hours.

AKI severity was underestimated because of fluid loading. Even a stage 3 decrease in GFR (a decrease by two thirds [9] by the RIFLE classification) failed to result in an increase in plasma creatinine during infusion in the simulated patient. The decrease in plasma creatinine concentration was substantial (approximately 20% for crystalloids and approximately 40% for colloids) irrespective of AKI severity. The rapid reduction in plasma creatinine during fluid boluses suggests that care must be taken in the timing of plasma sampling. If the bolus is large, sampling should be delayed at least 1 hour.

The majority of cardiac arrest patients demonstrated a creatinine concentration reduction that was greater than could be explained by dilution alone. We hypothesized that creatinine production ceased temporarily because shock brings a temporary halt to all metabolic processes. This could also explain a reduction in plasma cystatin C. The relative extent of change of creatinine and cystatin C depends on the differences in their volumes of distribution. The clinical consequences were that reliance on plasma creatinine underestimated the severity of AKI in the 5% of patients in whom AKI was detected (Cr_increase_), and failed to classify another 8% (Cr_unchanged_) as having AKI. As identified by Prowle and colleagues [27], who demonstrated that many cases of oliguria in the ICU are not associated with increased creatinine, 41 of the 44 patients not classified as AKI by plasma creatinine in this study developed oliguria during the first 48 hours. Injury biomarkers confirmed that in Cr_unchanged _there had been substantial renal injury despite an apparently normal plasma creatinine throughout the first 24 hours. These patients illustrate the new category of creatinine-negative, biomarker-positive AKI [28].

A possible explanation for poorer outcomes in patients with AKI when nephrology consultation is delayed is that continuing excess fluid administration results in creatinine dilution leading to an underestimation of the severity of the loss of renal function [29]. In a retrospective analysis of 253 patients with AKI, Macedo and colleagues [26] adjusted for creatinine dilution by multiplying the absolute creatinine by the proportional increase in total body water measured by the daily cumulative fluid balance. In 64 patients (25%), the adjusted creatinine reached the threshold for AKI (50% increase) at least one day earlier than the unadjusted creatinine. While clearly useful, this approach does not take into account the dynamic creatinine kinetics reported in this study, particularly with respect to timing of sampling and the reduction in creatinine excretion rate with increased dilution. In a prospective study, it was shown that nephrology intervention within 18 hours triggered by a plasma creatinine rise of 0.3 mg/dL reduced the subsequent peak creatinine concentration compared with standard practice [30]. Unfortunately, the absence of fluid data makes it difficult to decide whether this outcome resulted from the potential therapeutic effect of fluid loading or from dilution of the creatinine concentration [31]. Several goal-directed therapy trials under way may help to determine the therapeutic effect of fluid loading (see ClinicalTrials.gov registered trials NCT00510835, NCT00975793, NCT00306059, and NCT01654003).

In an ICU setting, the cost of adjusting for fluid balance and creatinine kinetics is minimal given that urine output is normally recorded and creatinine regularly sampled. For most of the Cr_decrease _patients, creatinine concentration decreased within 4 hours. We suggest that if plasma creatinine has not decreased after 4 hours in clinical practice, kidney injury biomarker(s) be measured. This obviates the expense of screening all patients and is probably within a clinically meaningful time frame with respect to early intervention.

To the best of our knowledge, the hypothesis that volume loading, often described as resuscitation, reverses loss of renal function in AKI remains to be tested in a randomized control trial. In a recent meta-analysis of goal-directed therapy versus standard fluid administration in surgery, improved renal outcomes after AKI could not be ascribed to administration of greater fluid volumes [32]. The conventional paradigm of fluid administration to maintain kidney perfusion in AKI has been challenged by evidence suggesting that even a small positive fluid balance contributes to increased mortality. Payen and colleagues [33] noted that mean positive fluid balance within 2 days of entry to the ICU was associated with increased mortality in patients with diagnosed AKI. Similarly, the percentage of fluid accumulation was lower in survivors than non-survivors both at AKI diagnosis and at peak creatinine in the study by Bouchard and colleagues [4]. Duration of fluid overload (> 10% fluid accumulation) was also associated with increased mortality. It was noted that patients with fluid overload had lower urine output and plasma creatinine at AKI diagnosis. Thus, a possible contributing cause to both of these findings is that patients with fluid overload on diagnosis of AKI had, on average, a greater loss of GFR than those without but that this was masked by dilution of plasma creatinine.

### Limitations

Because several parameters were not measureable in the case studies, the model relied on several assumptions. The distribution clearance was approximated to be 200 mL/minute, an expandable interstitial volume was assumed to be three times the plasma volume, and metabolic water production was assumed to be half that of the insensible losses (at 800 mL/day). The model was not sensitive to these parameters. We are unaware of any experimental or clinical studies that have measured rapid changes in creatinine production following cardiac arrest. There is experimental, but as yet no clinical, evidence that creatinine production may fall in sepsis by as much as 30% [34]. It is possible that, rather than a change in production, metabolic changes that prevent creatinine moving between compartments may be responsible for the phenomena observed. However, it should be noted that our definition of Cr_decrease _is simply a pattern recognition based on decreasing creatinine. The modeled change in GFR in each patient may be modified if they had loss of renal function as might be expected after a sudden loss of renal perfusion following cardiac arrest.

The model did not take into account potential increased capillary permeability, which is known to occur in sepsis and possibly during therapeutic hypothermia. Capillary leakage may reduce plasma volume; however, colloids (albumin) have been shown in patients with sepsis to increase the interstitial and plasma volumes more than the volume of infused fluids, possibly because of movement of fluid from the intracellular compartment [35]. On the other hand, resuscitation with cold fluids (crystalloids) appears to cause hypovolemia despite a positive fluid balance [36,37]. All of the case study patients underwent therapeutic hypothermia; none was septic. If increased capillary permeability had resulted in hypovolemia, the plasma concentrations would have been artificially elevated. We restricted analysis to cardiac arrests in order to minimize heterogeneity. Further study is required to see whether the results are generalizable to other forms of shock.

## Conclusions

The model accounts for fluid input and urine output and helps avoid misdiagnosis of AKI and AKI severity. Creatinine and volume kinetics were combined to quantify dynamic changes in GFR in the critically ill, providing insight into disease progression. The model enabled diagnosis of AKI in a cohort of cardiac arrest patients despite maintenance of a normal plasma creatinine concentration. This highlights that plasma creatinine alone cannot be relied upon to diagnose AKI after cardiac arrest and suggests caution in interpreting AKI epidemiology and trials that rely solely on plasma creatinine to define outcomes. This suggests that injury biomarkers are essential for identifying AKI in some clinical scenarios. Volume kinetic modeling could also enhance our understanding of the effects of fluid loading as a mediator of poor clinical outcomes [3] by quantifying the effects of fluid loading on different compartments.

## Key messages

• Dilution delays acute kidney injury (AKI) diagnosis.

• Avoid measurement of serum creatinine for 1 hour after a fluid bolus.

• AKI after cardiac arrest may be missed by relying on serum creatinine.

• A stable creatinine after cardiac arrest indicates AKI.

• Injury biomarkers are essential for identifying AKI in some scenarios.

## Abbreviations

AKI: acute kidney injury; ALP: alkaline phosphatase; Cr_decrease_: group of patients with a plasma creatinine decrease of more than 10% at 24 hours after cardiac arrest; Cr_increase_: group of patients with a plasma creatinine increase of more than 20% at 24 hours after cardiac arrest; Cr_unchanged_: group of patients not Cr_decrease _or Cr_increase_; ED: emergency department; GFR: glomerular filtration rate; GGT: gamma-glutamyltranspeptidase; GST: glutahione-S-transferase; ICU: intensive care unit; NGAL: neutrophil gelatinase-associated lipocalin; PCI: percutaneous coronary intervention; RIFLE: Risk, Injury, Failure, Loss, and End-stage kidney disease; ROSC: return of spontaneous circulation.

Creatinine kinetic model: C_1_: creatinine concentration in the plasma compartment; C_2_: creatinine concentration in the extracellular compartment; Ġ: creatinine generation rate; k_e_: rate constant for diffusion of creatinine to the plasma compartment from the extravascular compartment and vice versa (per minute); k_r_: rate constant for renal excretion of creatinine from the plasma (per minute); V_1_: volume of the plasma compartment; V_2_: volume of the extracellular compartment.

Volume kinetic model: Ḃ: fluid infusion rate; Cl_d_: distribution clearance (also known as k_t_); İ: insensible loss rate; Ṁ: metabolic production rate; $\dot{\text{U}}$: urine output rate; *v*_1_, *v*_2*e*_: expanded compartment volumes; V_10_: volume of the plasma compartment at time zero; V_2e0_: volume of the expandable compartment at time zero; V_2n0_: volume of the non-expandable compartment (constant).

## Competing interests

The authors declare that they have no competing interests.

## Authors' contributions

JWP conceived of, developed, and wrote the program for the model; co-designed the Early Detection of Acute Kidney Injury (EDAKI) study and carried out the data analysis and interpretation; and led the manuscript writing. AMdR collected the EDAKI data and participated in data interpretation and manuscript approval. ZHE participated in model development, co-designed the EDAKI study (principal investigator), and participated in data interpretation, manuscript writing, and approval. All authors read and approved the final manuscript.

## Supplementary Material

Additional file 1 **Extended methods and sensitivity analysis**. Details of the mathematical formulation of the combined creatinine and volume kinetic model. Sensitivity analysis of the model input variables: the rate of water distribution, the difference between insensible loss and metabolic water production, variations in the ratio of the plasma to expandable space, and creatinine generation. Click here for file
