# Supplementary material for: Combining creatinine and volume kinetics identifies missed cases of acute kidney injury following cardiac arrest
Source: Crit Care. 2013 Jan 17;17(1):R7. doi: 10.1186/cc11931 (PMC4056623; doi:10.1186/cc11931)
Supplement: Additional file 1 — Extended methods and sensitivity analysis. Details of the mathematical formulation of the combined creatinine and volume kinetic model. Sensitivity analysis of the model input variables: the rate of water distribution, the difference between insensible loss and metabolic water production, variations in the ratio of the plasma to expandable space, and creatinine generation. [file cc11931-S1.PDF]

Supplementary File to:

Combining creatinine and volume kinetics identifies missed cases of Acute Kidney Injury following Cardiac Arrest.

Pickering, J.W., Ralib, A.Md., Endre, Z.H.
September 2012.

Table of Contents

Complete methods 2
Creatinine kinetics 2
Volume kinetics 4
Combined creatinine kinetic and volume kinetic model 4
The Simulated Patient 5
Effect of fluid loading and differential rates of urine output 5
Urine output 6
Fluid input 8
Cases 9
Assays 10
Model Sensitivity Analysis. 10
Sensitivity analysis 1: distribution clearances 10
Sensitivity analysis 2: insensible losses 10
Sensitivity analysis 3: plasma volume to expandable volume 10
Sensitivity analysis 4: change in rate of creatinine production 10
References 10

Complete methods

A volume-creatinine kinetic model was developed by combining independent two-compartment models for each of creatinine kinetics and volume kinetics. The initial conditions of the model were assessed in a simulated patient. The model was then tested in a cohort of patients resuscitated after cardiac surgery. Three patients were presented as case studies.

Creatinine kinetics

The creatinine kinetic model is shown in Supplementary Figure 1A. Creatinine generated in muscle cells enters the extravascular compartment (V_2) at a rate $\dot{G}$, is freely exchanged with the plasma compartment (V_1), and is eliminated by the kidney at a rate determined by the rate constant, k_r ($Note\ GFR = k_r V_{10}$). We used dot-notation for differentiation (eg $\dot{G} = \frac{dG}{dt}$) and a subscript "0" to represent the value of the variable at time zero

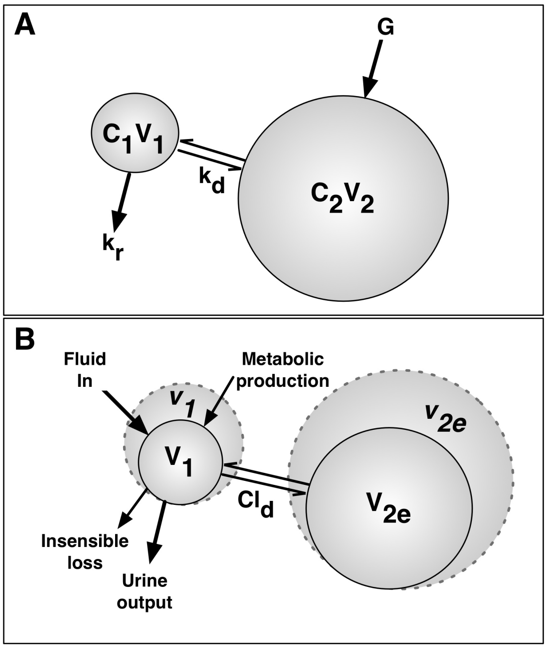

Supplementary Figure 1: Creatinine kinetic model (A) and Volume kinetic model (B).

It is assumed that the total volume of distribution of creatinine ($V_1 + V_2$) is equal to the total body water (TBW), the extravascular volume is equal to the total body water minus the plasma volume, and creatinine is not bound and flows freely from one compartment with the same permeability as water. The rate of change of mass in each compartment was determined by mass balance:

$$\begin{aligned}\dot{q}_1 = \ & gain\ from\ the\ extravascular\ compartment \\ & \quad - loss\ to\ the\ extravascular\ compartment \\ & \quad\quad - renal\ loss \\ = \ & k_{12}q_2 - k_{21}q_1 - k_r q_1\end{aligned} \tag{1}$$

Where the k's are the diffusion rate constants for creatinine and as the mass in each compartment is the product of the concentration and volume $q_1 = C_1V_1$ and $q_2 = C_2V_2$, ad

$$C_1\dot{V}_1 + \dot{C}_1V_1 = k_{12}C_2V_2 - k_{21}C_1V_1 - k_rC_1V_1 \quad (2)$$

As creatinine is not bound $k_{21} = k_{12} = k_d$. Rearranging equation 2 gives

$$\dot{C}_1 = \frac{\left[k_d(C_2V_2 - C_1V_1) - k_rC_1V_1 - C_1\dot{V}_1\right]}{V_1} \quad (3)$$

Applying the principal of mass balance to the extravascular compartment gives

$$\begin{aligned}\dot{q}_2 &= Generation\ rate + gain\ from\ the\ plasma\ compartment \\ &\qquad -loss\ to\ the\ plasma\ compartment \\ &= \dot{G} + k_{21}q_1 - k_{12}q_2\end{aligned} \quad (4)$$

Therefore,

$$C_2\dot{V}_2 + \dot{C}_2V_2 = G + k_{21}C_1V_1 - k_{12}C_2V_2 \text{ and} \quad (5)$$

$$\dot{C}_2 = \frac{\left[\dot{G} + k_d(C_1V_1 - C_2V_2) - C_2\dot{V}_2\right]}{V_2} \quad (6)$$

The creatinine generation rate may change with time. This may be modelled as a function of the initial generation rate, eg slowly reducing secondary to muscle wasting, or it may be modelled as discrete step changes.

At equilibrium (time zero) $\dot{C}_1 = \dot{C}_2 = \dot{V}_1 = \dot{V}_2 = 0, V_1 = V_{10}, V_2 = V_{20}, C_{10} = C_{20} = C_0, k_r = k_{r0}, \dot{G} = G_0$

Therefore, from equations 3, and 6

$$\begin{aligned}k_d &= \frac{k_{r0}V_{10}}{V_{20} - V_{10}} \\ &= \frac{G_0}{C_0(V_{20} - V_{10})}\end{aligned} \quad (7)$$

and

$$k_{r0} = \frac{G_0}{C_0V_{10}} \quad (8)$$

GFR ($= k_{r0}V_{10}$) may change with time (t). In this case the renal elimination rate constant changes with time,

$$k_r(t) = k_{r0}\big(1 - \Delta g(t)\big) \quad (9)$$

where

$$\Delta g(t) = \frac{GFR(0) - GFR(t)}{GFR(0)} \tag{10}$$

Volume kinetics

Volume kinetics simulates the distribution and elimination of infused fluids and usually involves two compartments, one which approximates the plasma volume (V_{10} prior to expansion), a second which is the expandable component of the V_2 fluid space (V_{2e}); Supplementary Figure 1B. Please note, in some volume kinetic literature these compartments will be labelled the central (V_c) and peripheral (V_t) body fluid spaces. V_{2e0} is smaller than the interstitial space because expansion of body fluids is not possible in all regions, for example, in the skeleton. As fluid is infused into the vasculature, the plasma compartment volume will expand at a rate dependent on the rate of infusion, $\dot{B}$, metabolic production, $\dot{M}$, insensible loss, $\dot{I}$, urine output, $\dot{U}$, and exchange between compartments. We assume water flow is the same in both directions between compartments given that fluid flows freely and does not bind to tissue and that there is no significant loss to a third space.

For each compartment:

$$Rate\ of\ volume\ expansion\ =\ Rate\ of\ fluid\ in\ -\ Rate\ of\ fluid\ out \tag{11}$$

Therefore,

$$\dot{V}_1 = \dot{B} + \dot{M} - \dot{I} - \dot{U} - \dot{T}\ , \tag{12}$$

$$\dot{V}_{2e} = \dot{T} \tag{13}$$

The rate of exchange between compartments, $\dot{T}$, depends on the distribution clearance (Cl_d) and the relative differences in the expansion of the two volumes:

$$\dot{T} = Cl_d\left(\frac{(v_1 - V_{10})}{V_{10}} - \frac{(v_{2e} - V_{2e0})}{V_{2e0}}\right) \tag{14}$$

V_{10} and V_{2e0} are the time zero compartment volumes, v_1 and v_{2e} are the time varying volumes. We assume water flux is the same in both directions between compartments and that there is no significant loss to a third space.

Combined creatinine kinetic and volume kinetic model

The creatinine and volume kinetic models were combined assuming that the creatinine kinetic plasma compartment and volume kinetic plasma volume were the same[1]. Let V_{2n0} be the non-expandable volume (a constant) equal to the difference between the initial extravascular and expandable interstitial space volumes ($V_{2n0} = V_{20} - V_{2e0}$). The extravascular compartment volume (V_2) is equal to the sum of the expandable and non-expandable volumes ($V_2 = v_{2e} + V_{2n0}$) and $\dot{V}_2 = \dot{V}_{2e}$. Substituting

volume kinetic variables for V_1 and V_2 in equation 3, gives the rate of change of plasma creatinine as a function of fluid volumes:

$$\dot{C}_1 = \left[k_d \left(C_2 (v_{2e} + V_{2n0}) - C_1 V_{10} \right) - k_r C_1 V_{10} - C_1 \left(\dot{B} + \dot{M} - \dot{I} - \dot{U} - Cl_d \left(\frac{(v_1 - V_{10})}{V_{10}} - \frac{(v_{2e} - V_{2e0})}{V_{2e0}} \right) \right) \right] / V_{10} \qquad (15)$$

The model was programmed in Matlab (Matlab 2011b, MathWorks Inc., Natick, MA, USA) and solved numerically with the ordinary differential equation solver *ode45*. Model nomenclature is given in Supplementary Table 1.

Supplementary Table 1. Model Nomenclature.

Creatinine Kinetic Model	
$\dot{G}$	Creatinine generation rate.
C_1	Creatinine concentration in the plasma compartment.
C_2	Creatinine concentration in the extracellular compartment.
V_1	Volume of the plasma compartment.
V_2	Volume of the extracellular compartment.
$q_1 = C_1 V_1$	Creatinine mass of the plasma compartment.
$q_2 = C_2 V_2$	Creatinine mass of the extracellular compartment.
k_r	Rate constant for renal excretion of creatinine from the plasma (min^{-1}).
k_{12} and $k_{21} = k_d$	Rate constant for diffusion of creatinine to the plasma compartment from the extravascular compartment and vice versa (min^{-1}).
Volume Kinetic Model	
$\dot{I}$	Insensible loss rate.
$\dot{M}$	Metabolic production rate.
$\dot{B}$	Fluid infusion rate.
$\dot{U}$	Urine output rate.
$\dot{T}$	The net rate of fluid exchange between two compartments. This depends both on the fluid (colloid or crystalloid) and (is proportional to) the deviation from equilibrium of each compartment[2].
V_{10}, V_{2e0}	Initial (time zero) volumes of the plasma and expandable extravascular compartments.
v_1, v_{2e}	Expanded compartment volumes (vary with time).
V_{2n0}	The non-expandable extravascular volume (a constant at all times)
Cl_d	Distribution clearance (sometimes in the literature as k_t).

The Simulated Patient

Effect of fluid loading and differential rates of urine output

The change in creatinine concentration and fluid balance as a function of time was calculated for a 70kg male with total body water of 42L, plasma volume of 2.8L, expandable interstitial volume of 8.4L[1], baseline GFR of 100ml/min, baseline creatinine concentration of 1mg/dl, and initial creatinine generation rate of 1mg/min

reducing by 1.5% per day. Simulated patient demographics are given in Supplementary Table 2.

Supplementary Table 2: Simulated patient demographics

Ideal total body water	ITBW (ml)	42,000
Initial plasma volume	V_{10} (ml)	2800 (ITBW/15)
Initial expandable space volume	V_{2e0} (ml)	8,400 (3*ITBW/15)
Initial creatinine generation rate	$\dot{G}_0$ (mg/min)	1
Glomerular filtration rates	GFR	100, 66.7, 50, 33.3
Insensible water loss rate	$\dot{I}$ (ml/day)	800
Metabolic water generation rate	$\dot{M}$ (ml/day)	400
Distribution clearance rate	Cl_d (ml/min)	200, 10
Initial plasma creatinine concentrations	$C_{10} \equiv C_{20}$ (mg/dl)	1

Urine output

The rate of urine output depends on hydration status. In someone with normal kidney function, extreme hypertonic dehydration (loss of 10% of total body water) results in an approximate 70% reduction in urine output. Over-hydration will produce a very rapid increase in urine output, peaking at no more than about 900ml/h assuming normal kidney function. We suggest that as little as a 2.5% increase in total body water will result in a 10-fold increase in urine output. In the initially euhydrated patient, urine output will rapidly increase on administration of fluids. In a dehydrated patient, water is initially conserved resulting in a delay before urine output increases. Urine output is the product: $Urine\ output = GFR \times (100 - Reabsorption\ rate)/100$. The reabsorption rate also changes with GFR, resulting in proportionally greater reabsorption when GFR decreases assuming the mechanisms remain intact. For example, at 100% hydration when the GFR is normal there is 99% reabsorption resulting in 1% of GFR as the rate of urine output. When GFR is halved, the reabsorption rate increases to 99.5% and urine output is reduced by 75%. Using the initial conditions of Supplementary Table 3 the reabsorption rate can be described by the formula:

$$Reabsorption\ rate = 99.7e^{-0.0000109(Hydration\ -90)^{2.81}} \quad <100\%\ \text{hydration}$$
$$Reabsorption\ rate = 1.30\times10^{-9}(120 - Hydration\ )^{7.71} + 85 \quad \geq100\%\ \text{hydration} \qquad (16)$$

Supplementary Table 3: Urine output boundary conditions

Hydration (Percentage normal body weight)	Reabsorption rate	Urine output change	Hourly urine output example (ml/hr)
100%	99%	Reference	60
102.5%	90%	1000%	600
120%	85%	1500%	900
90%	99.7%	30%	18
95%	99.6%	40%	24

Equation 16 is illustrated graphically in Supplementary Figure 2.

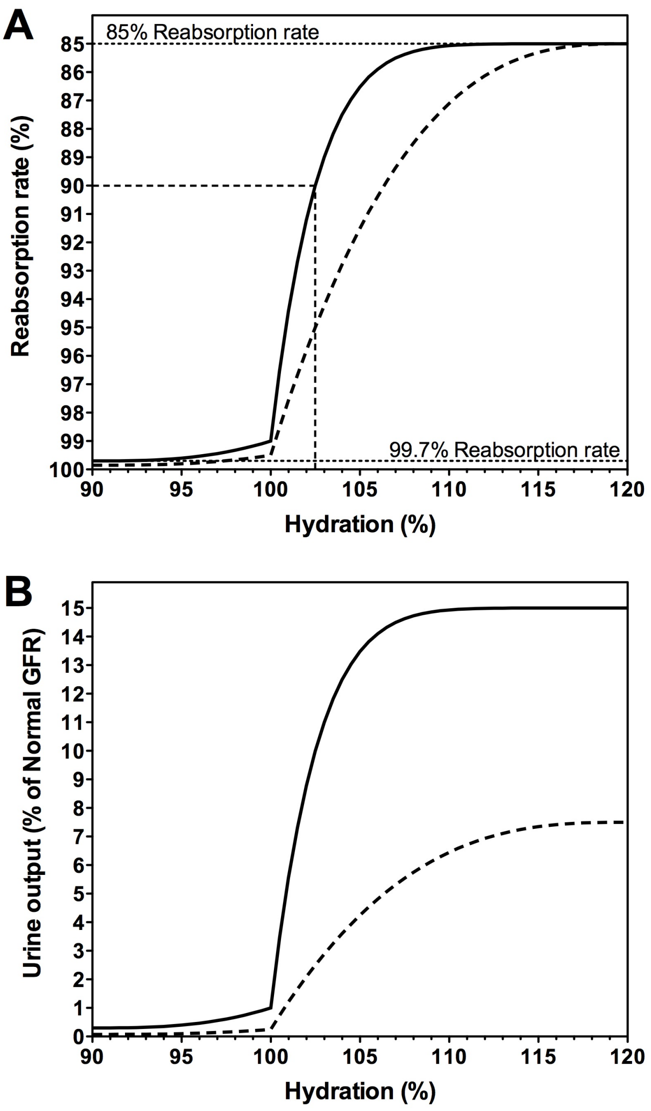

Supplementary Figure 2: Modelled change in tubular reabsorption rate (A) and urine output (B) according to hydration status. Maximum rate of reabsorption is 99.3% at 90% hydration equivalent to a reduction in urine output of 70% from normal hydration where reabsorption is 99%. Reabsorption decreases rapidly with over-hydration to 90% (10-fold increase in urine output rate) at 102.5% and reaches a minimum of 85% resulting in a maximal 15-fold increase in urine output (ie to approximately 900 ml/h for a person with urine output of 60 ml/h when normally hydrated). The dashed curve shows the reabsorption rate and urine output for when GFR is 50% of normal.

Supplementary Figure 3 illustrates the urine output for a simulated patient with the following characteristics: 70kg male (TBW = 42L) who receives a maintenance fluid and a bolus and has GFR=100 ml/min, insensible loss = 800 ml/day (0.556 ml/min) and metabolic water production 400 ml/day (0.278 ml/min). The maintenance fluid equals the normal urine output at normal GFR plus the total insensible loss less the metabolic water production:

$$Maintenance\ fluid\ (ml/min) = 0.99 * GFR\ (ml/min) + insensible\ loss - metabolic\ water\ production$$

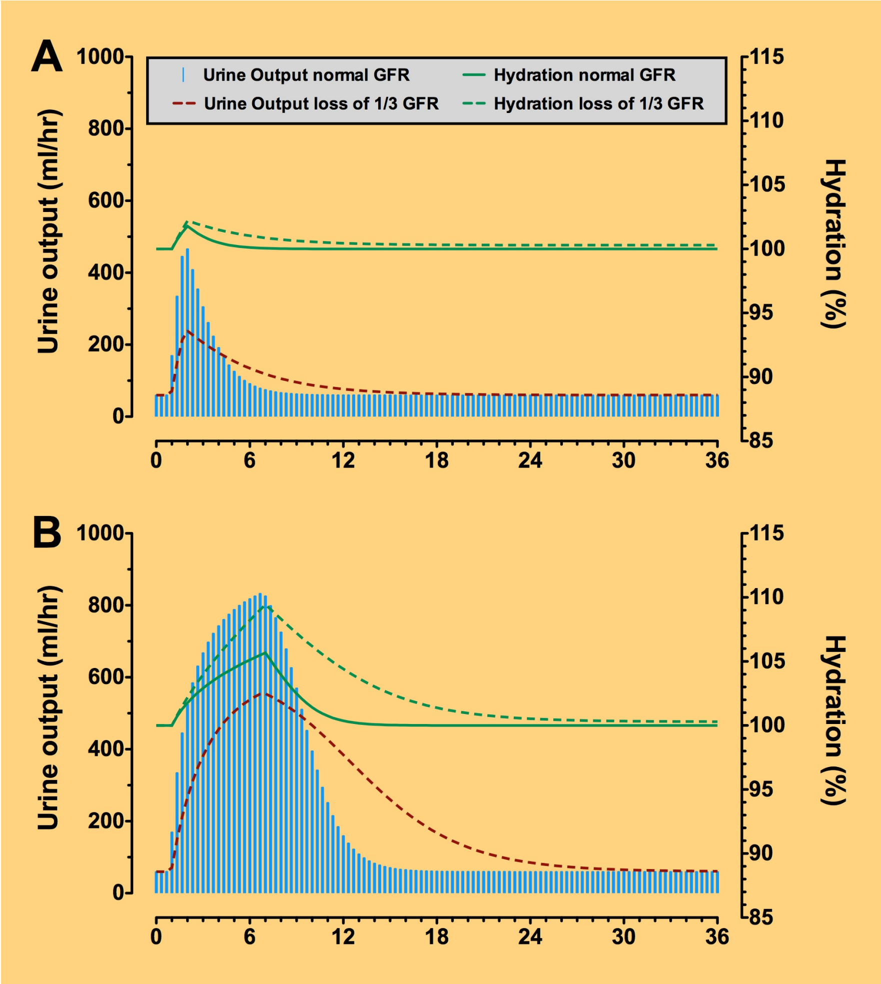

Supplementary Figure 3: Urine output (ml/hr) for the model patient with normal GFR (blue) and one third loss of GFR at hour one (red dashed) following a bolus of 1 L over 1 hour (A) and 6 L over 6 hours (B) starting at hour one. Hydration percentages are shown as solid green (normal GFR) and dashed green (1/3rd loss of GFR). Maximum hydration reached for normal GFR /1/3rd loss GFR models (hour): A, 101.8/102.2% (2:00); B, 105.7/109.4% (7:00).

Fluid input

Two fluid input scenarios were modelled: maintenance fluid alone, and maintenance + boluses of either crystalloids or colloids of one litre per hour for six hours beginning at the end of hour one. This represents a high fluid input and corresponded to the 80th percentile of the fluid input over the first 6 hours of the patients in the cardiac arrest cohort. For each fluid input scenario there were four GFR scenarios: no change in GFR, a decrease by one third, one half, or two-third's at the time of insult (t=1h). These correspond to GFR criteria for RIFLE AKI severity stages R (Risk), I (Injury), and Failure (F). On the basis of creatinine-kinetic modelling alone these produce increases of creatinine of 50%, 100% and 200% respectively.

The crystalloid distribution exchange rate (Cl_d) was set at 200ml/min. This was based on multiple studies with Ringers lactate (Supplementary Table S4). Colloids have much longer half-lives in the vasculature, and correspondingly smaller distribution exchange rates. We set Cl_d for colloids at 10ml/min.

Metabolic water production was assumed to be 400 ml/day (16.7ml/h) and insensible losses 800ml/day (33.4ml/h). At a GFR of 100 ml/min and 99% reabsorption, urine output was 1 ml/min (60ml/h). Therefore maintenance fluid was set at 76.7ml/h ($Maintenance = \dot{U} + (\dot{I} - \dot{M})$).

Supplementary Table S4 Measured distribution rate constant, Cl_d

Cl_d (ml/min)	Fluid	# subjects	Subjects	Reference
43 (33-63)	Ringer's acetate	8	Female, pregnant, healthy	[3]
100 (60-127)	Ringer's acetate	8	Female, pregnant, Pre-eclampsia	[3]
116 (46-161)	Ringer's acetate	10	Female, not pregnant, healthy	[4]
79 (38-144) (Arterial samples)	Ringer's lactate	15	8 Female, 7 Male, healthy	[5]
51 (25-150) (Venous samples)	Ringer's lactate	15	8 Female, 7 Male, healthy	[5]
320 and 147	Ringer's acetate	2	Male, healthy	[2]
148 (88-208)	Ringer's acetate	6	Female, healthy	[6]
195±51	Ringer's acetate	10	Male, healthy	[7]
214±32	Ringer's acetate	10	Male, healthy – mildly hypovolemic (450ml blood withdrawn)	[7]
192±22	Ringer's acetate	10	Male, healthy– hypovolemic (900ml blood withdrawn)	[7]
208 (166-385)	Ringer's acetate	10	Male, healthy	[8]
164 (143-251)	Ringer's lactate	10	Male, healthy	[8]
168 (124-426)	Normal Saline	10	Male, healthy	[8]
169±19	Normal Saline	12	5 Female, 7 Male, healthy	[9]
207±48	Ringer's acetate	10	5 Female, 5 Male, Prior to spinal anaesthesia	[10]
186±31	Ringer's acetate	10	6 Female, 4 Male, Prior to general anaesthesia	[10]
173±27	Ringer's acetate	9	Male, healthy	[11]
115 (61-227)	Ringer's acetate	12	Undergoing elective laparoscopic cholecystectomy	[12]
60 (50-74)	7.5% Saline	10	Male, healthy	[8]

Data expressed as mean±standard error of the mean, or median (lower quartile – upper quartile)

Cases

Patients admitted following cardiac arrest were a subcohort from the Early Detection of Acute Kidney Injury (EDAKI) study conducted in the emergency department (ED) and intensive care unit of Christchurch Hospital (ACTRN12610001012066). Ethics approval was obtained from The Upper South A Regional Ethics Committee (URA/09/09/062) and informed consent was obtained from patients or relatives.

After ICU admission each patient immediately underwent therapeutic hypothermia for 24h (core temperature reduced to 33°C). The resuscitation fluid was 0.9% saline in each case prior to arrival at the ED. Hourly urine output and fluid input were recorded for each patient. Plasma samples were collected on admission to the emergency department, on admission to the ICU and at least daily thereafter. Urine samples for measurement of neutrophil-gelatinase-associated lipocalin (NGAL), cystatin C, alkaline phosphatase (ALP), gamma-glutamyltranspeptidase (GGT), and α- and π-glutahione-S-transferase (α- and π-GST), were taken on catheterisation in the emergency department, on admission to the ICU, 4, 8, 16h later and daily.

In addition to assessing the general characteristics of this cohort, examples of patients with complete data from each of the three groups shown in figure 3 of the manuscript were selected for intensive analysis. The first male enrolled in each group was selected.

Patient A was a 90kg male, with a history of hypertrophic obstructive cardiomyopathy and with severely impaired left ventricular function (ejection fraction 25%). He had presented to ED complaining of abdominal pain, nausea and vomiting. His condition deteriorated requiring intubation, during which time he suffered a cardiac arrest followed by cardio-pulmonary resuscitation (CPR). He was defibrillated twice (at 360J) and adrenaline given. After 50min he regained cardiac output and was transferred to the ICU. Over the next 4 days, his cardiopulmonary status improved steadily, however there was no neurological recovery, presumably due to hypoxic brain injury. Therapy was withdrawn and he passed away.

Patient B was a 87kg male, with a history of alcoholism. He collapsed during a shower. Resuscitation time was 20 minutes with marginal CPR He received 1.5L of normal saline in the ED and another 1L in the first two hours in the ICU. Prognosis was assessed as poor after rewarming. Multiple fluid boluses were given, nevertheless the patient became oliguric and passed away 53 hours post-arrest.

Patients C was a 80kg, male, with no previous medical history. He suffered a cardiac arrest during a motor vehicle inspection. Immediate CPR was performed by a nurse bystander before the arrival of paramedics. Resuscitation lasted 27min with defibrillation (once) and two adrenaline boluses. An electrocardiograph showed an inferolateral myocardial infarct, and the patient underwent emergency percutaneous coronary intervention with a stent inserted to repair the left circumflex artery that was completely occluded. He was transferred to the ICU for cooling. There were several occasions of bradycardia needing boluses of atropine and later adrenaline infusion. After rewarming his condition improved and he was extubated. He was discharged to a coronary care unit on day 3.

Assays

Plasma and urine creatinine were measured by the Classical Jaffe Reaction on Architect c8000 analyzer using Abbott reagents (Abbott Laboratories, Abbott Park, Illinois, USA). Plasma and urine cystatin C were measured by particle enhanced

nephelometric immunoassay on a BNII nephelometer (Dade Behring GmbH, Marburg, Germany). Plasma NGAL was analysed using the The Triage® NGAL Test (Alere, San Diego, USA). Urine AP and GGT were measured on Architect c8000 analyzer using Abbott reagents (Abbott Laboratories, Abbott Park, Illinois, USA) by p-nitrophenol and γ-glutamyl-p-nitroanilide rate reactions respectively. Urine NGAL was also measured on the Architect platform. Urine α- and π-GST were assayed using enzyme immunoassays (Argutus Medical Alpha GST EIA-BIO-91 and Pi GST EIA-BIO85, Argutus Medical, Dublin, Ireland).

Model Sensitivity Analysis.

The control is the simulated patient modelled plasma creatinine distribution following boluses of 1000 ml/h for 6 hours using the estimates for the distribution clearance (Cl_d = 200 ml/min), insensible loss rate (800 ml/day) and ratio of plasma volume to expandable interstitial volume (1:3) used with the case studies. Supplementary Table 5 gives the initial values for each sensitivity analysis. The sensitivity analysis for the distribution clearance ranged from 25% (50 ml/min) to 200% (400 ml/min) of the control (see Supplementary Table 3 for published values), for the insensible loss rate from 50% (400 ml/day) to 150% (1200 ml/day), plasma volume to expandable interstitial volume ratio from 1:2 (interstitial volume of 5600 ml) to 1:4 (11200 ml), and changes in the creatinine generation rate from 0.7 to 1.3 mg/min.

Supplementary Table 5: Virtual-In-Patient characteristics for the sensitivity analyses

	Sensitivity analysis 1: $Cl_{d,}$	Sensitivity analysis 2: Insensible loss	Sensitivity analysis 3: Expandable volume	Sensitivity analysis 4: Creatinine generation
Weight, kg	70	70	70	70
Total Body Water, L	42	42	42	42
Baseline creatinine, mg/dl	1	1	1	1
Creatinine generation rate, mg/min	1	1	1	**0.7, 1, 1.3**
GFR, ml/min	100	100	100	100
Initial hydration status, %	100	100	100	100
Fluids, ml/h	1000 for 6h	1000 for 6h	1000 for 6h	1000 for 6h
$Cl_{d,}$ ml/min	**50, 100, 200, 300, 400**	200	200	200
Insensible loss, ml/day	800	**400, 800, 1200**	800	800
Metabolic production, ml/day	400	400	400	400
Plasma volume, ml	2800	2800	2800	2800
Expandable volume, ml	8400	8400	**5600, 8400, 11200**	8400

Sensitivity analysis 1: distribution clearances

The model was insensitive to changes in distribution clearance rates with less than 10% deviation; Supplementary Figure 4. This is comparable to the coefficient of variation of many plasma creatinine assays.

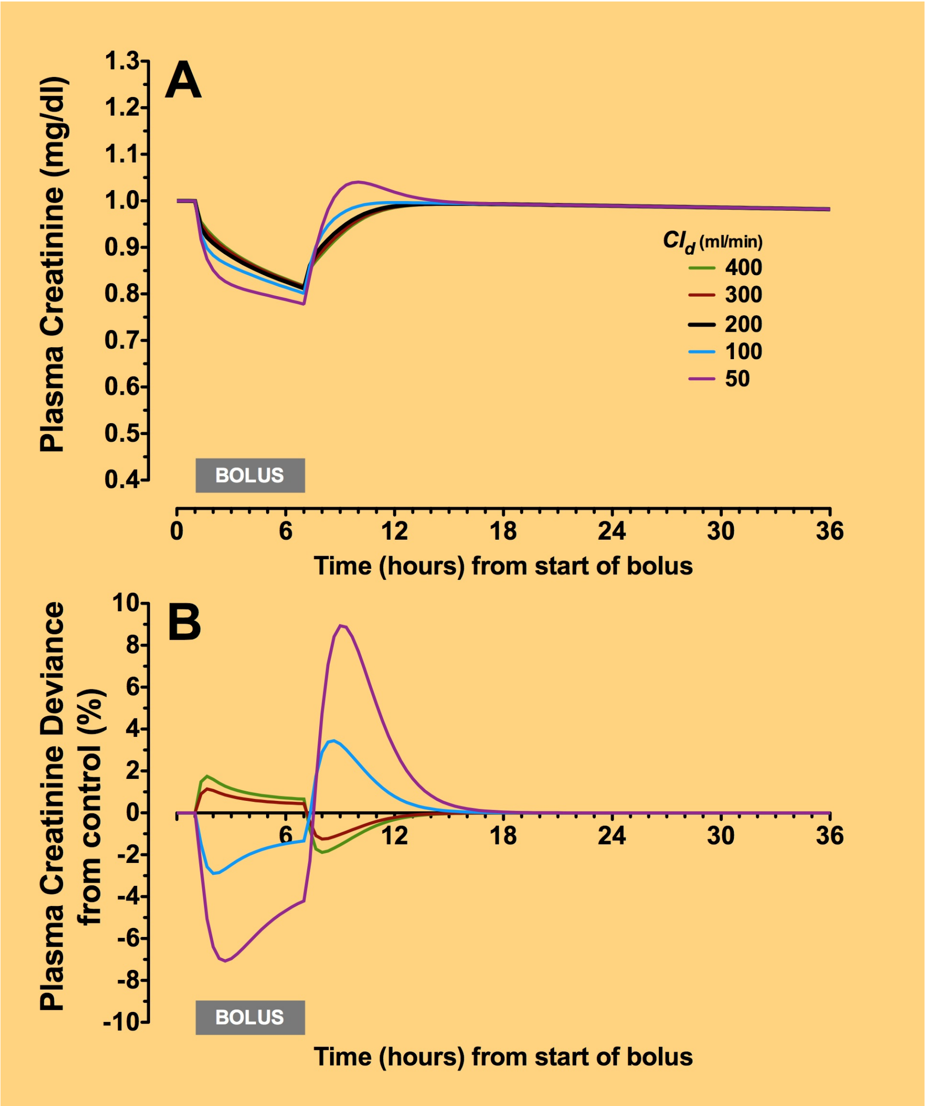

Supplementary Figure 4: Sensitivity Analysis 1: (A) Plasma creatinine changes for distribution clearances (Cl_d) varying from 5 to 400 ml/min in model patient with 6L Bolus. (B) Deviations from the control (Cl_d = 200 ml/min) concentrations for Cl_d ranging from 50 to 400 ml/min. Maximum deviation 8.9% (Cl_d = 50 ml/min).

Sensitivity analysis 2: insensible losses

The model was not sensitive to a ±50% change in insensible loss whilst maintaining the same metabolic production rate; Supplementary Figure 5.

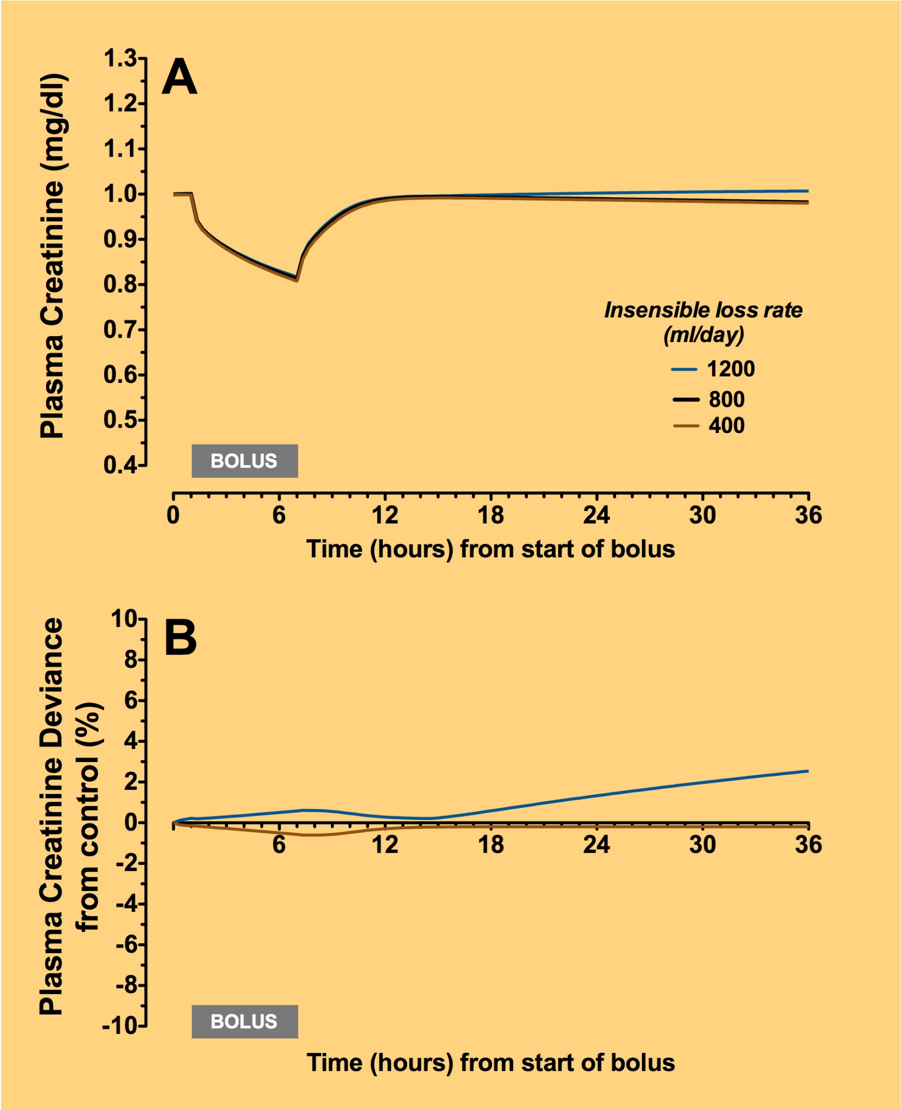

Supplementary Figure 5: Sensitivity Analysis 2: (A) Plasma creatinine changes for insensible loss rates of 400, 800 and 1200 ml/day. (B) Deviations from the plasma creatinine control (800 ml/day) concentrations for the model with insensible loss rates of 400 ml/day and 1200 ml/day. Maximum deviation 4.7% (1200 ml/day).

Sensitivity analysis 3: plasma volume to expandable volume

The model was not sensitive to large variations in the ratio of the plasma (V_{10}=2800ml) to expandable volume (V_{2e0}varying from 5600 ml to 11200 ml); Supplementary Figure 6.

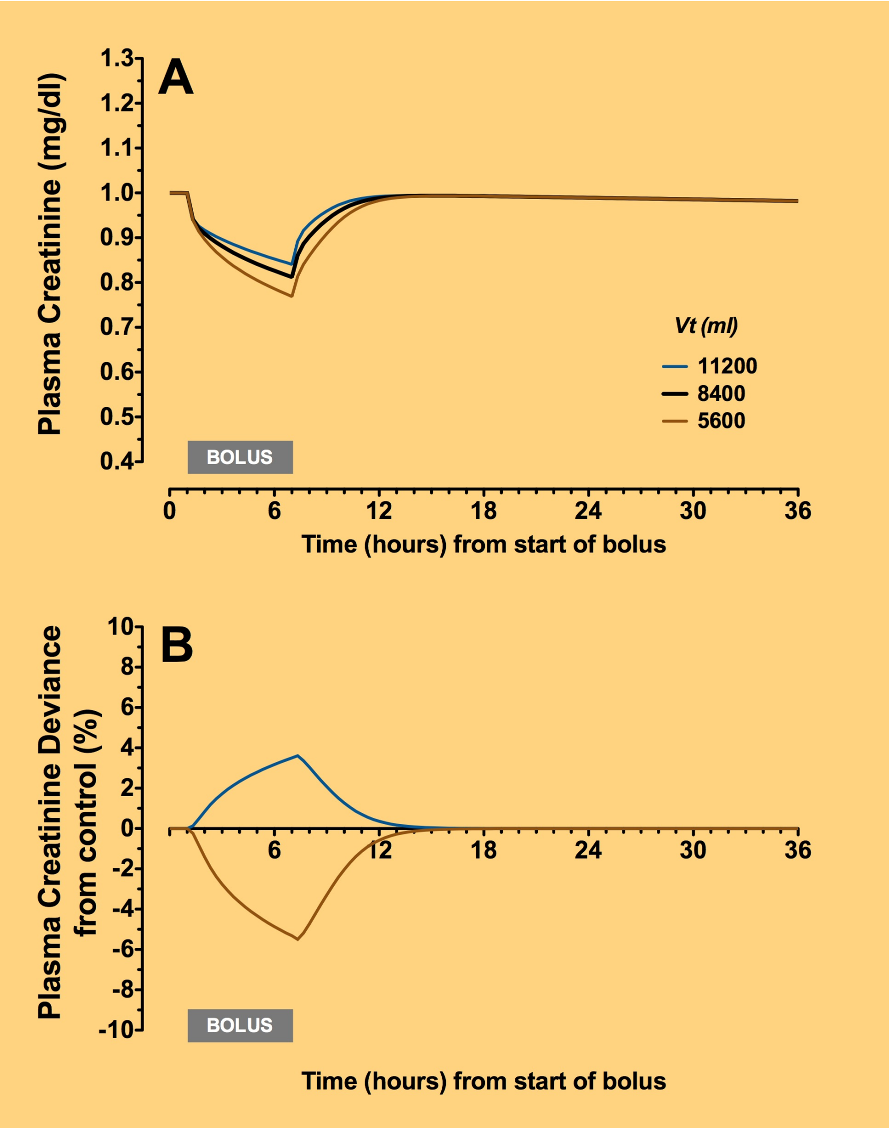

Supplementary Figure 6: Sensitivity Analysis 3: (A) Plasma creatinine changes for expandable volumes of 5600, 8400 and 11200 ml with a plasma volume of 2800ml in all cases. (B) Deviations from the plasma creatinine control (8400 ml) concentrations for models with expandable volumes of 5600 ml (1:2 ratio) and 11200 ml (1:4 ratio). Maximum deviation -5.5% (5600 ml).

Sensitivity analysis 4: change in rate of creatinine production

The model was sensitive to a brief (1h), or sustained (36h) ±30% change in plasma creatinine production; Supplementary Figure 7.

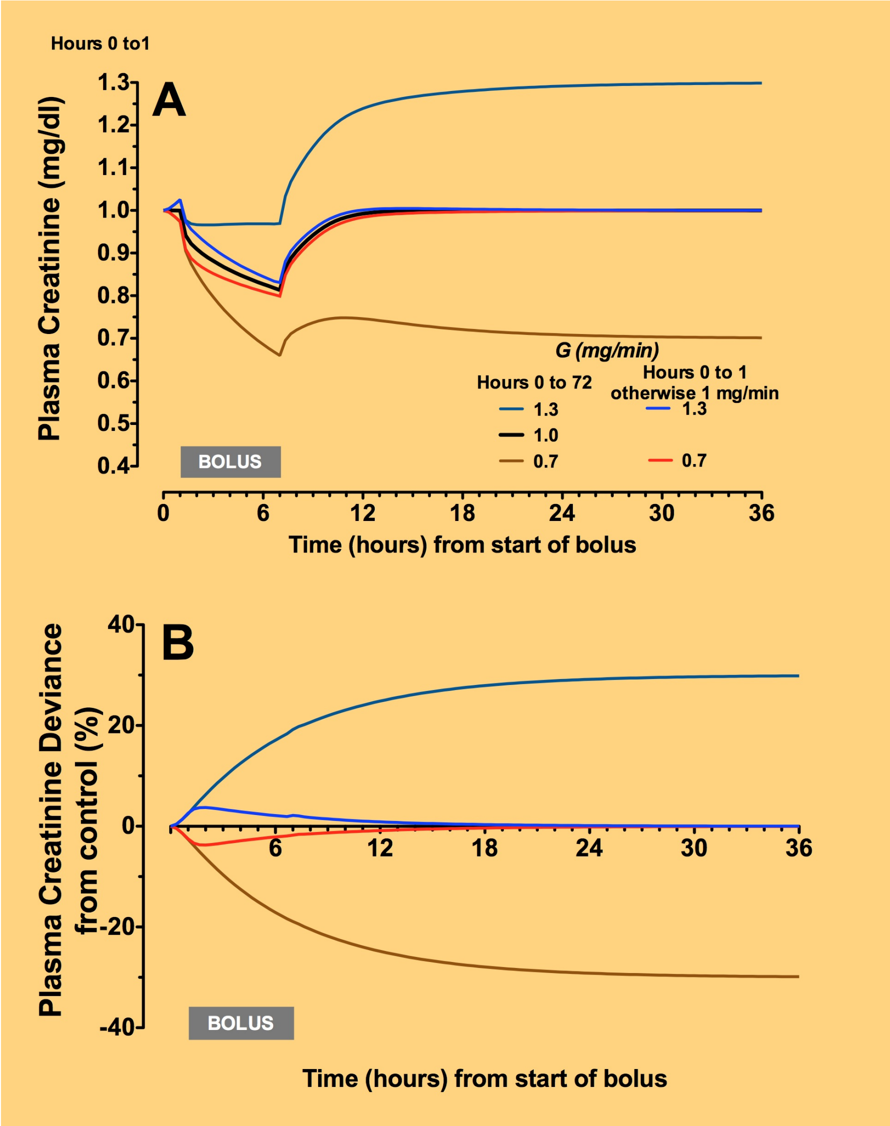

Supplementary Figure 7: Sensitivity Analysis 4: (A) Plasma creatinine generation rate declined to 70% or 130% of control (1 mg/min) and the decline was maintained for either 1 hour (Hours 0 to 1) or 36 hours (Hours 0 to 36). (B) Percentage deviations from the control.

References

1. Hahn RG: **Volume kinetics for infusion fluids**. *Anesthesiology* 2010, **113**:470–481.

2. Ståhle L, Nilsson A, Hahn RG: **Modelling the volume of expandable body fluid spaces during i.v. fluid therapy**. *Br J Anaesth* 1997, **78**:138–143.

3. Drobin D, Hahn RG: **Distribution and elimination of crystalloid fluid in pre-eclampsia**. *Clin Sci* 2004, **106**:307–313.

4. Rodhe P, Drobin D, Hahn RG, Wennberg B, Lindahl C, Sjostrand F, Svensen CH: **Modelling of peripheral fluid accumulation after a crystalloid bolus in female volunteers - a mathematical study**. *Computational and mathematical methods in medicine* 2010:1.

5. Svensen CH, Rodhe PM, Olsson J, Børsheim E, Aarsland A, Hahn RG: **Arteriovenous differences in plasma dilution and the distribution kinetics of lactated ringer's solution**. *Anesth Analg* 2009, **108**:128–133.

6. Hahn R, Drobin D, Stahle L: **Volume kinetics of Ringer's solution in female volunteers**. *Br J Anaesth* 1997, **78**:144–148.

7. Drobin D, Hahn R: **Volume kinetics of Ringer's solution in hypovolemic volunteers**. *Anesthesiology* 1999, **90**:81–91.

8. Drobin D, Hahn R: **Kinetics of isotonic and hypertonic plasma volume expanders**. *Anesthesiology* 2002, **96**:1371–1380.

9. Norberg A, Hahn RG, Li H, Olsson J, Prough DS, Borsheim E, Wolf S, Minton RK, Svensen CH: **Population volume kinetics predicts retention of 0.9% saline infused in awake and isoflurane-anesthetized volunteers**. *Anesthesiology* 2007, **107**:24–32.

10. Ewaldsson C, Hahn R: **Volume kinetics of Ringer's solution during induction of spinal and general anaesthesia**. *Br J Anaesth* 2001, **87**:406–414.

11. Sjostrand F, Edsberg L, Hahn R: **Volume kinetics of glucose solutions given by intravenous infusion**. *Br J Anaesth* 2001, **87**:834–843.

12. Olsson J, Svensen C, Hahn R: **The volume kinetics of acetated Ringer's solution during laparoscopic cholecystectomy**. *Anesth Analg* 2004, **99**:1854–1860.
